# Transcatheter Correction of Bilateral Partial Anomalous Pulmonary Venous Return with Intrapulmonary Dual Drainage: A Rare Entity

**DOI:** 10.3390/life16020316

**Published:** 2026-02-12

**Authors:** Dusan Andric, Andrija Pavlovic, Igor Stefanovic, Marko Pavlovic, Maja Trkulja, Maja Bijelic, Milica Kuzmanovic, Jovan Petrovic, Mirko Topalovic, Vojislav Parezanovic, Milan Djukic

**Affiliations:** 1 Department of Cardiology, University Children’s Hospital, 11000 Belgrade, Serbia; 2 Medical Faculty, University of Belgrade, 11000 Belgrade, Serbia; 3 Institute for Cardiovascular Diseases Dedinje, 11000 Belgrade, Serbia; 4 University Medical Centre Ljubljana, 1000 Ljubljana, Slovenia

**Keywords:** partial anomalous pulmonary venous return, dual drainage, transcatheter correction, vascular plug

## Abstract

Partial anomalous pulmonary venous return (PAPVR) with dual drainage is a very rare congenital heart anomaly. We report the case of a 6-year-old boy with PAPVR in whom both upper pulmonary veins (PVs) drain anomalously into the systemic venous circulation, while maintaining preserved intrapulmonary collateral venous connections with the remaining pulmonary veins draining into the left atrium. Careful balloon occlusion testing of the anomalous PVs was performed, simultaneously with measurements of pulmonary pressures and control angiography, proving the absence of venous congestion in the upper lung fields during the pulmonary venous phase. Transcatheter occlusion using vascular plugs was safely and successfully performed.

## 1. Introduction

Partial anomalous pulmonary venous return (PAPVR) occurs when one or more, but not all, pulmonary veins (PVs) drain into the systemic venous circulation. Left-sided PVs typically connect anomalously to the left innominate vein (IV) via a persistent embryological vessel, known as the vertical vein (VV), or, less commonly, to the coronary sinus. Right-sided PVs typically connect to the superior vena cava (SVC) or inferior vena cava (IVC) [1]. In most cases, anomalous PVs connect to these systemic veins and lack a normal connection to the left atrium [2].

In this case report, we describe a rare variant of PAPVR in which the right upper pulmonary vein (RUPV) drains directly into the SVC, while the left upper pulmonary vein (LUPV) is connected to the IV via a VV. Both PVs have a preserved intrapulmonary collateral venous connection to the left atrium [3].

## 2. Case Report

A 6-year-old boy was evaluated at our outpatient clinic of a tertiary care university center because of a systolic murmur and mildly reduced exertion. Apart from recurrent respiratory infections, the remaining history was unremarkable. Transthoracic echocardiography (TTE) demonstrated a patent foramen ovale, without evidence of significant shunt, and confirmed normal drainage of the right lower pulmonary vein (RLPV). However, the right upper pulmonary vein was not clearly visualized. The left PVs were not visualized, but a VV with a diameter of about 6 mm was identified, draining pulmonary venous blood into the brachiocephalic vein with unobstructed flow. The SVC was dilated with increased flow velocity. The right-sided cardiac chambers were enlarged, with moderate tricuspid valve insufficiency. After discussion in the combined pediatric cardiology and surgical meeting, the consensus was to proceed with diagnostic cardiac catheterisation and possible transcatheter closure.

After routine preparation, we performed right heart catheterization under general anesthesia via a transfemoral approach. Oximetry samples were obtained from the SVC, IVC, and main pulmonary artery (MPA). The pressures obtained by direct manometry are expressed as three measurements: systolic, diastolic, and mean pressure (Table 1).

**Figure 1:**
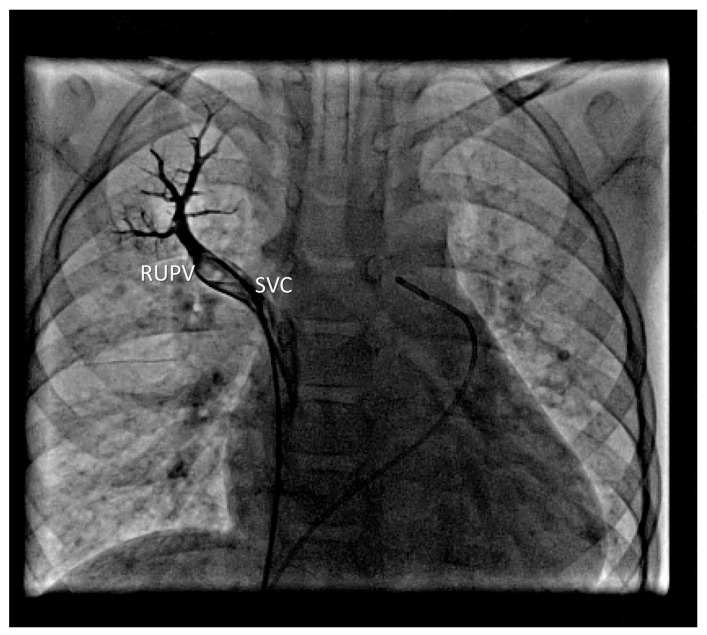
Selective venography of anomalous RUPV.

A pigtail catheter was introduced into the SVC and then advanced into a VV. When venography was performed, pressures were measured in MPA and its branches, and samples for oximetry were obtained. Pulmonary angiography from the MPA showed normal arborization of the pulmonary arteries (PA). In the venous phase, anomalous pulmonary venous return was visualized, and the LUPV drained via a large VV. Selective angiography of the left PA revealed normal drainage of the left lower pulmonary vein (LLPV). Selective angiography of the right PA showed anomalous drainage of the RUPV directly into the SVC, just before it entered the right atrium (Figure 1).

**Figure 2:**
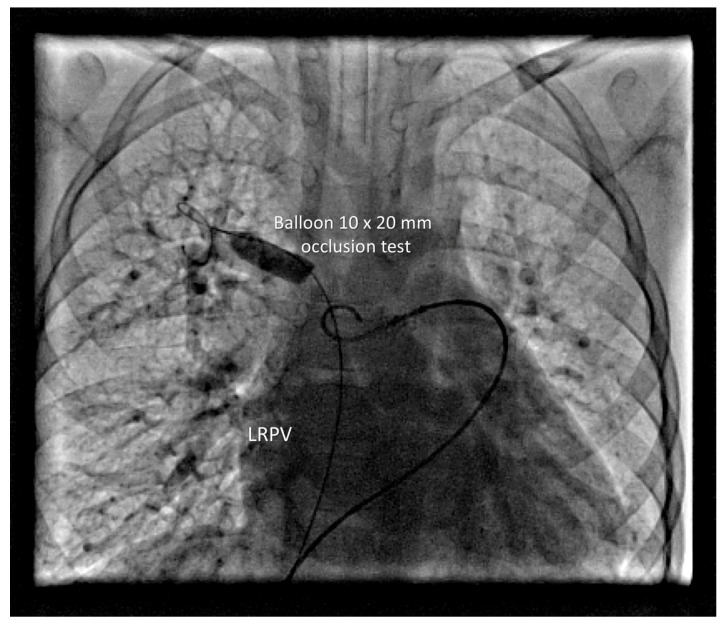
Balloon test occlusion of the RUPV.

**Figure 3:**
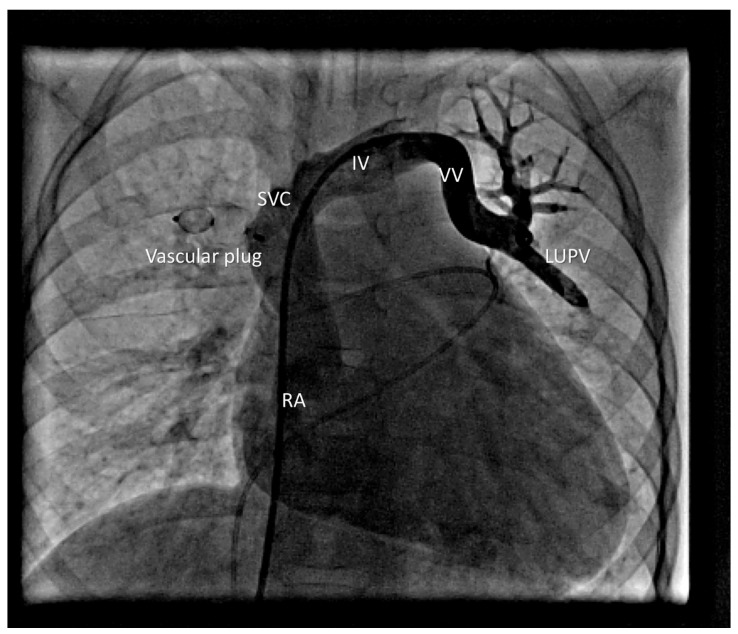
Vascular plug in RUPV without right upper pulmonary lobe congestion. Anomalous LUPV drains via VV and IV into the SVC.

The RLPV drained normally. A 5 Fr sheath was placed in the left femoral vein. Balloon occlusion testing was performed using a 10 mm × 20 mm balloon in RUPV and LUPV, followed by selective left and right PA angiography, respectively (Figure 2). No residual anomalous venous pathways were visualized in either upper pulmonary lobe. Pulmonary venous return drained freely and rapidly into the left atrium, with no significant increase in MPA pressure during prolonged balloon occlusion. A 12 mm Amplatzer Vascular Plug II (Abbott Medical, Plymouth, USA) was deployed in the RUPV (Figure 3).

**Figure 4:**
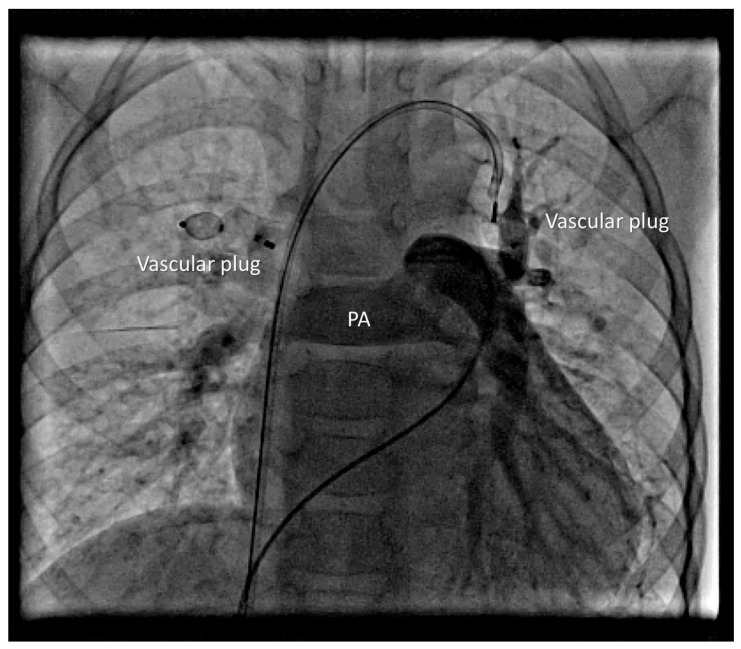
Vascular plug in the LUPV.

Device position was verified, and control angiography showed no venous congestion in the right upper lobe before release. A 7 Fr sheath was then placed in the left femoral vein, and a 7 Fr right guiding catheter was advanced into the VV. A 12 mm Amplatzer Vascular Plug I (Abbott Medical, Plymouth, USA) was delivered. After verifying the correct position and excluding pulmonary venous congestion, the device was released (Figure 4).

Final pulmonary angiography showed no residual anomalous venous drainage. A mild increase in MPA pressure was noted. Following the occlusion of the VV and the RUPV, a decrease in venous oxygen saturation is observed in the SVC and the MPA (Table 1). The procedure was completed without complications, aside from transient contact-related arrhythmias.

At the two-year follow-up, the patient had a single prolonged respiratory infection one-month post-procedure, resolving completely without complications. TTE shows normal right-sided heart dimensions without pulmonary hypertension (PH), physiological tricuspid valve regurgitation, and normal movements of the interventricular septum. Chest X-ray showed a normal pulmonary vascular pattern without pulmonary congestion.

Our case describes the safety and feasibility of percutaneous closure of PAPVR and the positive impact on hemodynamic and chamber measures.

## 3. Discussion

The prevalence of PAPVR in the pediatric population is less than 1% [4]. The hemodynamic consequences are directly correlated with the number of anomalous PVs and the size of the ASD. Children with PAPVR are usually asymptomatic, which suggests that the actual prevalence may be higher than reported. The natural course of the disease is similar to that of left-to-right shunt anomalies. Although congestive heart failure may rarely develop during childhood, the main consequence of excessive pulmonary blood flow is pulmonary hypertension (PH), typically occurring in the third and fourth decades of life [5].

For the classification of PV anomalies, adequate knowledge of the embryological development of the pulmonary vascular bed (PVB) and PVs is essential. The PVB develops from a portion of the splanchnic plexus. During early gestation, the primitive lungs drain into the systemic circulation through the vitelline, umbilical, and cardinal venous systems. At the beginning of the seventh week of gestation, a solid structure known as the common pulmonary vein separates from the dorsal mesocardium, establishing a connection between the splanchnic venous plexus and the primitive cardiac tube.

With the gradual formation of a lumen within the common pulmonary vein, it becomes incorporated into the posterior wall of the left atrium, between the right and left horns of the sinus venosus, to the left of the septum primum and superior to the coronary sinus. Following the establishment of this communication, the original connections between the pulmonary and systemic venous systems regress. In the presented case, the anomalous pulmonary venous connection resulted from a disturbance in the embryogenesis of the common pulmonary vein [6].

Alsoufi et al. classified PAPVR into 5 types: (1) right PAPVR to the SVC, (2) right PAPVR to the right atrium, (3) right PAPVR to the IVC, (4) left PAPVR to the left IV via ascending VV, and (5) bilateral PAPVR. Of note, this classification did not include a dual pulmonary venous connection [7]. Patel et al. described the concept which includes “extrapulmonary” and “intrapulmonary” separation of the dual drainage [8,9]. Therefore, our patient can be categorized as having Alsoufi type 5 (bilateral PAPVR) combined with Patel’s intrapulmonary dual drainage type.

PAPVR is commonly associated with an ASD, most frequently of the superior sinus venosus type. In our patient, only a small defect was observed in the region of the fossa ovalis. The clinical course of PAPVR is often insidious, with patients remaining asymptomatic or only minimally symptomatic for many years. In cases with a significant left-to-right shunt, the natural history of the condition includes progressive pulmonary vascular remodeling. While these changes are initially reversible, they may become irreversible by the third decade of life, ultimately resulting in the development of PH.

The definition of PH in children has traditionally mirrored that of adults, with a mean pulmonary arterial pressure (mPAP) ≥ 20 mmHg [10]. The 6th World Symposium on Pulmonary Hypertension (6th WSPH) proposed a revised definition of PH in adults as mPAP > 20 mmHg, along with the inclusion of pulmonary vascular resistance (PVR) ≥ 3 WU for the identification of pre-capillary pulmonary hypertension. In pediatric PH, particularly in the context of congenital heart disease, it is recommended to assess PVR indexed (PVRI) to body surface area in order to evaluate the presence of PH, which is defined as PVRI ≥ 3 WU/m^2^ [11].

PAPVR is a silent congenital cardiac anomaly. In cases where TTE demonstrates right heart enlargement without an identifiable cause, it is essential to consider the possibility of anomalous PV drainage and to pursue further diagnostic evaluation. One of the main limitations of TTE is its inability to reliably visualize the entire interatrial septum and detect anomalous PVs. Elevated oxygen saturation levels in the SVC are indicative of a left-to-right shunt and support the diagnosis of PAPVR [8,12,13]. In our case, RHC revealed an oxygen saturation of 96% in the SVC prior to the site of the shunt. The oxygen saturation in the SVC before shunt is higher than usual because anomalous PVs carrying oxygenated blood from the lungs drain into the SVC, which normally transports deoxygenated blood from the upper extremities and the head, resulting in blood mixing. For the same reason, oxygen saturation in the MPA is higher than expected. However, after occlusion of the anomalous PVs, mixing no longer occurs, resulting in a decrease in oxygen saturation within the MPA (Table 1). A potential concern during transcatheter closure of PAPVR is passive hyperemia and an increase in pressure within the pulmonary vascular bed. Therefore, during the procedure, a balloon occlusion test is routinely performed to monitor any rise in pressure within the MPA. To assess the hemodynamic tolerance of redirecting anomalous PVs return into the left atrium, an Amplatzer sizing balloon was temporarily inflated within the anomalous venous pathway and maintained for approximately 10 min. During balloon occlusion, continuous direct manometry hemodynamic monitoring was performed with particular attention to pulmonary venous pressures in order to detect any significant increase. The absence of a significant or sustained rise in pulmonary venous pressure and mPAP during the occlusion period indicated that the pulmonary circulation could safely tolerate redirection of flow, thereby supporting the feasibility and safety of definitive transcatheter closure. In our case, only a minimal increase in mPAP was observed (4 mmHg). Closure should not be performed if a mPAP rises ≥ 10 mmHg [14].

Intrapulmonary venovenous collaterals are considerably less common in PAPVR than extrapulmonary collaterals [8,9]. Aggarwal et al. emphasize that they are most frequently observed in patients with univentricular physiology, particularly after the Glenn or Fontan procedure, where chronically elevated systemic venous and transpulmonary pressures promote their development as a vent-like mechanism to decompress the pulmonary vascular bed [8]. A similar mechanism likely explains the development of collateral channels between the venous systems of the upper and lower lung fields in our case. Drainage of the lower lung fields into the left atrium was anatomically preserved, with both inferior PVs demonstrating normal and adequate drainage.

The concept of duplicated intrapulmonary venous drainage proposed by Patel et al. cannot be reliably demonstrated using angiography alone [9]. Consequently, balloon test occlusion was performed prior to definitive closure to assess the hemodynamic impact of vessel occlusion. The absence of an increase in MPA pressure during test occlusion indicated sufficient decompression of the pulmonary vascular bed, likely mediated by collateral vessels within the pulmonary parenchyma. By plugging the systemic connection, blood is effectively redirected through the existing intrapulmonary collateral pathways toward the left atrium, thereby re-establishing a more physiological circulation without the need for open-heart surgery.

In the case reported by Wilson et al., dual drainage of PAPVR was extrapulmonary [14]. Angiography via a transjugular approach demonstrated that the LUPV drained into the IV through a VV while maintaining a direct connection to the left atrium. Following test balloon occlusion of the VV, no significant rise in proximal venous pressure was observed, allowing safe device closure. This finding supports the feasibility of transcatheter occlusion in selected cases of dual-drainage PAPVR when adequate PVs return to the left atrium is preserved.

At present, there is a growing experience of percutaneous transcatheter device closure. It offers many advantages over surgical treatment, such as fast recovery, avoidance of mechanical ventilation, pediatric cardiac intensive care unit stay, short in-hospital stay, low cost, and more patient satisfaction.

In this case, occlusion was performed using Amplatzer vascular plugs type I and type II, both self-expanding devices made of braided nitinol mesh. Type I has a single-lobe design, while type II features a triple-lobe configuration to enhance occlusive performance. Device selection depends on the target vessel diameter, with oversizing of 30–50% recommended. Both devices are effective in occluding arteriovenous fistulas, aortopulmonary collaterals, Blalock–Taussig shunts, and fenestrations in Fontan circulation, and, as demonstrated in this case, they can also be used to occlude venous vessels [15].

## 4. Conclusions

Although surgery is the treatment of choice, transcatheter correction may be a feasible alternative in carefully selected patients with intrapulmonary or extrapulmonary dual drainage of anomalous PVs.

The Amplatzer Vascular Plug I and II are good choices for occlusion of anomalous venous vessels of this type (VV and RUPV in our case) via a transfemoral approach.

## Figures and Tables

**Table 1:** Oximetry and manometry findings during right heart catheterization.

	Pressure Before Occlusion (mmHg)	Pressure After Occlusion (mmHg)	SpO_2_ Before (%)	SpO_2_ After (%)
SVC	/	/	96	89
IVC	/	/	86	/
MPA	29/15/21	31/18/25	94	86
PVs	/	/	99	/

SVC—superior vena cava, IVC—inferior vena cava, MPA—main pulmonary artery, PVs—pulmonary veins, and SpO_2_—oxygen saturation.

## Data Availability

The original contributions presented in this study are included in the article/Supplementary Materials. Further inquiries can be directed to the corresponding author.

## Supplementary Materials

The following supporting information can be downloaded at: https://www.mdpi.com/article/10.3390/life16020316/s1. Captions: Video S1. Selective entry into the left superior pulmonary vein which flows into the innominate vein via the vertical vein. Video S2. Balon test occlusion of the vertical vein. Video S3. Selective entry into the right superior pulmonary vein. Video S4. Balon test occlusion of the right superior pulmonary vein. Video S5. Vascular plug in the right superior pulmonary vein and delivery cable with vascular plug in the left superior pulmonary vein.

## Abbreviations

The following abbreviations are used in this manuscript:

PAPVR	Partial Anomalous Pulmonary Venous Return
PVs	Pulmonary Veins
IV	Innominate Vein
VV	Vertical Vein
SVC	Superior Vena Cava
IVC	Inferior Vena Cava
RUPV	Right Upper Pulmonary Vein
LUPV	Left Upper Pulmonary Vein
RLPV	Right Lower Pulmonary Vein
MPA	Main Pulmonary Artery
PA	Pulmonary Arteries
LLPV	Left Lower Pulmonary Vein
TTE	Transthoracic Echocardiography
PH	Pulmonary Hypertension
PVB	Pulmonary Vascular Bed
